# Linking Resting-State Networks in the Prefrontal Cortex to Executive Function: A Functional Near Infrared Spectroscopy Study

**DOI:** 10.3389/fnins.2016.00452

**Published:** 2016-10-07

**Authors:** Jia Zhao, Jiangang Liu, Xin Jiang, Guifei Zhou, Guowei Chen, Xiao P. Ding, Genyue Fu, Kang Lee

**Affiliations:** ^1^School of Computer and Information Technology, Beijing Jiaotong UniversityBeijing, China; ^2^Department of Applied Psychology and Human Development, Dr. Eric Jackman Institute of Child Study, University of TorontoToronto, ON, Canada; ^3^Department of Computer Science, University College LondonLondon, UK; ^4^Department of Psychology, Hangzhou Normal UniversityHangzhou, China; ^5^Department of Psychology, Zhejiang Normal UniversityJinhua, China; ^6^Department of Psychology, National University of SingaporeSingapore, Singapore

**Keywords:** resting-state, fNIRS, small-world, executive function, prefrontal cortex

## Abstract

Executive function (EF) plays vital roles in our everyday adaptation to the ever-changing environment. However, limited existing studies have linked EF to the resting-state brain activity. The functional connectivity in the resting state between the sub-regions of the brain can reveal the intrinsic neural mechanisms involved in cognitive processing of EF without disturbance from external stimuli. The present study investigated the relations between the behavioral executive function (EF) scores and the resting-state functional network topological properties in the Prefrontal Cortex (PFC). We constructed complex brain functional networks in the PFC from 90 healthy young adults using functional near infrared spectroscopy (fNIRS). We calculated the correlations between the typical network topological properties (regional topological properties and global topological properties) and the scores of both the Total EF and components of EF measured by computer-based Cambridge Neuropsychological Test Automated Battery (CANTAB). We found that the Total EF scores were positively correlated with regional properties in the right dorsal superior frontal gyrus (SFG), whereas the opposite pattern was found in the right triangular inferior frontal gyrus (IFG). Different EF components were related to different regional properties in various PFC areas, such as planning in the right middle frontal gyrus (MFG), working memory mainly in the right MFG and triangular IFG, short-term memory in the left dorsal SFG, and task switch in the right MFG. In contrast, there were no significant findings for global topological properties. Our findings suggested that the PFC plays an important role in individuals' behavioral performance in the executive function tasks. Further, the resting-state functional network can reveal the intrinsic neural mechanisms involved in behavioral EF abilities.

## Introduction

Executive function (EF) refers to a set of higher order psychological processes that are involved in goal-oriented behavior (Zelazo and Müller, 2002). It consists of a variety of cognitive components, such as planning, working memory, short-term memory, inhibition, and switch (Zelazo and Müller, 2002; De Luca et al., 2003; Testa et al., 2012). EF plays vital roles in our everyday adaptation to the environment. Executive dysfunction may increase the risk of serious cognitive or social problems, such as attention-deficit hyperactivity disorder (ADHD), autism, and Parkinson's disease (Zelazo and Müller, 2002; Miyasaki et al., 2006).

Due to EF's significant role in our lives, its neural correlates have been extensively investigated in many neuropsychological, clinical, and neuroimaging studies. Researches have consistently shown that the prefrontal cortex (PFC) is involved in EF. The most direct evidence comes from neuropsychological studies, suggesting that cerebral lesions or damages to the PFC cause deficits in EF (Owen et al., 1990, 1991; Burgess et al., 2000; Bonnì et al., 2014). Clinical studies have also shown that mental illnesses with EF deficits, such as obsessive-compulsive disorder (OCD), depression, and schizophrenia are related to functional impairment of PFC (Selemon et al., 1998; Davidson et al., 1999; Gu et al., 2008; Melloni et al., 2012). Additionally, recent neuroimaging studies using a variety of non-invasive functional neuroimaging methodologies, such as functional magnetic resonance imaging (fMRI), magnetoencephalography (MEG), and functional near-infrared spectroscopy (fNIRS), have revealed significant relations between EF performance and neural activation levels in the PFC in both healthy and clinical individuals (Peters et al., 2009; Pu et al., 2011; Melloni et al., 2012; Moriguchi and Hiraki, 2013; de Vries et al., 2014; Oh et al., 2014). For example, for healthy participants, fMRI activation in the bilateral PFC was associated with a planning task (Newman et al., 2003), and the neural MEG activities can be enhanced by a set-shifting task (Oh et al., 2014). For patients with some mental illnesses (e.g., Alzheimer's disease or depression), the decreased neural activities of the PFC was related to a poor performance of EF tasks (Peters et al., 2009; Pu et al., 2011).

However, it should be noted that the existing neuroimaging studies mostly focused on the activation of the PFC elicited by EF tasks. Limited existing studies have linked EF to the resting-state brain functional activity. Resting-state functional activity refers to the internally spontaneous fluctuations of the brain in a natural condition without stimulation (Biswal et al., 1995). It has been consistently shown that functional connectivity in the resting state between the sub-regions of our brains can reveal the intrinsic neural mechanisms involved in cognitive processing without disturbance from external stimuli (Fox et al., 2005).

The human brain is a dynamical system that is characterized by complex exchanges of information among the brain regions (Friston, 1994). Such dynamic interaction and synergy of multiple brain regions is necessary for high level cognitive processing, especially executive function (Douw et al., 2011). Indeed, some recent studies have found significant relations between high level cognitive processing and functional connectivity. For example, Wang et al. (2011) found that the level of intelligence was correlated with resting-state connectivity of multiple brain regions including the middle frontal, inferior parietal lobules. Douw et al. (2011) using MEG reported significant correlations between the overall brain resting-state topology and performance in cognitive tasks measuring EF, attention, and working memory. Additional studies have also demonstrated that resting-state functional connectivity abnormality may be associated with cognitive decline (Damoiseaux et al., 2008; Douw et al., 2011; Wang et al., 2011). Most relevant to the present study, Reineberg et al. (2015) found that EF was associated with functional connectivity intensity of the right frontoparietal resting-state functional network identified by independent components analysis (ICA). This finding suggested that the resting-state functional connectivity analysis method can be applied to the study of neural correlates of EF.

The present study used resting-state high-density fNIRS to examine the relations between behavioral performances in different EF tasks and functional connectivity of the PFC. fNIRS is a non-invasive neuroimaging methodology that measures cortical hemodynamic changes optically (Villringer and Dirnagl, 1994; Tak and Ye, 2014). It has many advantages over fMRI (Ding et al., 2013; Tak and Ye, 2014) which are particularly important for the present study. Over the past decade, the appropriateness of using fNIRS to examine localized cortical neural activities has been well established (Ferrari and Quaresima, 2012; for reviews, see Boas et al., 2014). In particular, systematic studies with human adults using both fNIRS and fMRI have produced highly consistent results regarding the cortical locations of specific cognitive functions (for a review, see Cui et al., 2011). Although the spatial resolution of NIRS is worse than MRI (cm vs. mm), the temporal resolution of fNIRS is greater than that of fMRI (e.g., 10 vs. 0.5 Hz). The higher temporal resolution of fNIRS is helpful to describe the time course of the fluctuations of the brain's neural activities more precisely. More specifically, the high temporal resolution of the fNIRS signals allows for preventing the higher frequency physiological signals from interfering with low-frequency fluctuations (Lu et al., 2010), which is the focus of our study. Additionally, the operating cost and complexity of a NIRS system is far less than a MRI machine, allowing for the collection of data from a larger sample of participants to ensure a high level of statistical power. The advantages of fNIRS over fMRI lend fNIRS very well as a tool to study functional connectivity.

The applications of resting-state fMRI have been widely accepted (for a review, see Lee et al., 2013). Given the similarity between fNIRS and fMRI hemodynamic signals (Steinbrink et al., 2006), it is reasonable to extend the techniques of resting-state study to fNIRS. Over the past several years, there were an increasing number of studies on the resting-state functional connectivity using fNIRS (for a review, see Niu and He, 2014). For example, Lu et al. (2010) obtained the resting-state fNIRS functional connectivity maps using both seed-based correlation analysis and data-driven cluster analysis. Niu et al. (2012) investigated the topological organization of the brain functional networks based on fNIRS, and found that the topological properties were consistent with previous fMRI findings. Recently, Li et al. (2015) capitalized on fNIRS' higher temporal resolution and examined the dynamic characteristics of the resting-state functional connectivity on the whole cortex by a sliding-window correlation method. They revealed high temporal variabilities in the cortical resting-state functional connectivity. In summary, the feasibility and validity of fNIRS for the assessment of resting-state neural activities had been sufficiently established.

The present study capitalized on the advantages of fNIRS to obtain resting-state functional data from 90 adults. We then used graph theory to analyze the resting-state data. The analysis of the complex brain networks based on graph theory is one of the most widely used methods in resting-state functional connectivity analysis (for a review, see van den Heuvel and Pol, 2010). This is because graph theory provides the state-of-the-art measures to describe the interaction and synergy of information between the brain regions from a global perspective. In the present study, we used this method to link the network properties of the functional connectivity among the regions of the PFC to behavioral EF performances.

Although some studies have linked the resting-state functional connectivity to performances in various EF tasks (Widjaja et al., 2013; Lin et al., 2015; Reineberg et al., 2015), to the best of our knowledge, few have specifically focused on the resting-state network topological properties. One exception was an MEG study by Douw et al. (2011). They, however, only focused on global parameters of resting-state network and did not analyze regional parameters. Thus, it is entirely unclear whether and to what extent the nodal and global topological properties of the brain network were associated with behavioral performances in various EF tasks. The present study aimed to bridge this significant gap in the literature.

More specifically, we aimed to examine an important theoretical question that hitherto has yet to be answered empirically: Whether and to what extent are global or local neural network connectivities associated with executive functioning in behavior? One possibility is that both types of connectivities are important to engender EF behaviors and therefore the greater people's global and local functioning connectivities, the greater their behavioral EF performance (the Global and Local Property Hypothesis). Although no specific evidence exists to support this possibility, a recent study showed that the resting-state global neural connectivity indexes are predictive of participants' intelligence as measured by IQ (Langer et al., 2012). Given some components of EF (e.g., working memory) is part of an intelligence scale that measures IQ, we hypothesized that global connectivity indexes are related to EF performance. Further, given the roles of the PFC in EF processing revealed by the existing studies, we hypothesized that the local topological properties of the resting-state brain functional network in the PFC would be significantly correlated with participants' overall EF performance. In addition, given the fact that different EF components (e.g., working memory, switch) entails different cognitive processes with different underlying neural mechanisms, we hypothesized that participants' scores in specific EF components would be related to different regional properties and such linkage should emerge in different regions in the PFC.

However, it is well established in the behavioral literature that IQ is an index of a general and global intellectual ability (van den Heuvel et al., 2009; Langer et al., 2012), whereas EF is a specialized cognitive ability (though with wide-range usages in a variety of situations). Thus, an alternative possibility is that unlike IQ, EF might be more associated with local than global neural connectivities (the Local Property Only Hypothesis). If this possibility is true, the local, but not global, network topological properties would be expected to link to both overall EF behavioral performance and different EF components.

## Materials and methods

### Participants

Ninety healthy right-handed young adults (30 males; 20.4 ± 1.5 years old) with normal or corrected to normal vision participated in the present study. None of them had any history of learning disabilities, neurological and psychiatric disorders. All participants gave informed written consent prior to their participation. This research was approved by the Ethics Committee of Zhejiang Normal University.

### Imaging acquisitions and data preprocessing

During the resting state, participants were required to sit still with eyes closed but not fall asleep, and to think of nothing as far as possible. The NIRS data collection lasted 12 min for each participant.

A 24 channel continuous wave system (ETG-4000 Hitachi Medical Co., Japan) was used for resting-state fNIRS data acquisition. The instrument consisted of five light emitters (each generated two wavelengths of near-infrared light: 760 and 850 nm) and four detectors on each hemisphere which allowed for 24 different measurement channels. During the experiment, the probes were embedded in two rubber shells, which were covered with a swimming cap to keep it attached to the participant's head. The inter-optode distance was 30 mm and the sampling rate was set to 10 Hz. The measurement of neural activities approximately 15–25 mm beneath the scalp was achieved.

**Figure 1:**
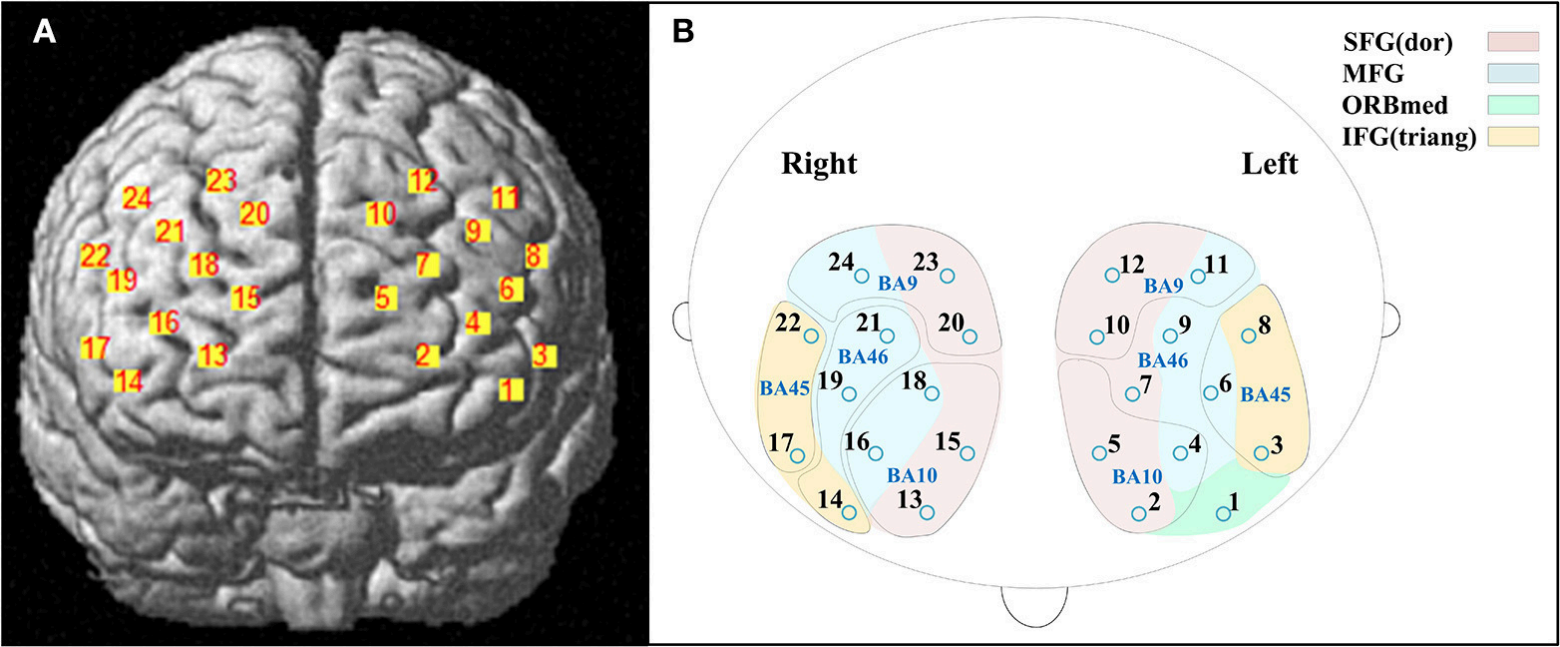
**Locations of near-infrared spectroscopy (NIRS) channels**. **(A)** The estimated positions of the NIRS channels. **(B)** The anatomical label (color) and Brodmann's Areas (MRIcro) (curve). Abbreviation: SFG (dor), dorsal superior frontal gyrus; MFG, middle frontal gyrus; ORBmed, Orbitofrontal cortex (medial); IFG (triang), triangular inferior frontal gyrus.

A 3D digitizer (EZT-DM401, Hitachi Medical Corporation, Japan) was used to complete the 3 dimensional spatial registration of NIRS channel locations. The estimated corresponding location of each NIRS channel in the Montreal Neurological Institute (MNI) space was obtained using the probabilistic registration method (Singh et al., 2005). The NIRS channels covered the bilateral PFC, including the dorsal superior frontal gyrus (SFG), the middle frontal gyrus (MFG), the triangular inferior frontal gyrus (IFG) and the left orbital MFG (Brodmann's Areas 9, 10, 45, 46) (Figure 1). The Brodmann's Areas (MRIcro), anatomical label and MNI coordinates were listed in Table 1.

**Table 1:** **The Brodmann's Areas (BA), anatomical label (AAL), and MNI coordinates of near-infrared spectroscopy (NIRS) channels**.

**Channel**	**BA**	**AAL**	**MNI (x, y, z)**
1	46	ORBmed	−46, 54, −3
2	10	SFG (dor)	−26, 68, 3
3	45	IFG (triang)	−53, 41, 5
4	10	MFG	−38, 60, 12
5	10	SFG (dor)	−17, 71, 17
6	45	MFG	−47, 46, 21
7	46	SFG (dor)	−27, 61, 26
8	45	IFG (triang)	−52, 29, 28
9	46	MFG	−37, 47, 34
10	9	SFG (dor)	−14, 58, 39
11	9	MFG	−44, 31, 43
12	9	SFG (dor)	−23, 45, 46
13	10	SFG (dor)	27, 70, 4
14	46	IFG (triang)	46, 58, −2
15	10	SFG (dor)	18, 71, 18
16	10	MFG	39, 63, 12
17	45	IFG (triang)	54, 44, 6
18	10	MFG	28, 63, 26
19	46	MFG	47, 50, 21
20	9	SFG (dor)	16, 60, 37
21	46	MFG	36, 51, 34
22	45	IFG (triang)	54, 33, 29
23	9	SFG (dor)	25, 48, 46
24	9	MFG	44, 35, 43

*SFG (dor), dorsal superior frontal gyrus; MFG, middle frontal gyrus; ORBmed, Orbitofrontal cortex (medial); IFG (triang), triangular inferior frontal gyrus*.

The raw optical signal was firstly converted to hemoglobin signal using the modified Beer-Lambert Law conducted by NIRS-SPM (Ye et al., 2009). To obtain relatively steady signals, the first 2-min of data for each participant were discarded. Preprocessing was conducted by the resting-state fMRI data analysis toolkit (REST) (http://resting-fmri.sourceforge.net). Briefly, after removing the linear trend, in order to reduce low-frequency drift and high-frequency physiological noise, the hemoglobin data were passed through a band-pass filter (0.009–0.08 Hz) which was consistent with previous fNIRS study (Niu et al., 2012). Although both oxygenated hemoglobin ([oxy-Hb]) and deoxygenated hemoglobin ([deoxy-Hb]) signals were obtained in this study, we only chose [oxy-Hb] data to perform further analyses due to its superior signal-to-noise ratio relative to [deoxy-Hb] (Strangman et al., 2002; Homae et al., 2007).

### Behavioral tasks description

After the image acquisitions, the participants were measured EF behaviorally using the computer-based Cambridge Neuropsychological Test Automated Battery (CANTAB). CANTAB is a widely used and well validated cognitive test software system that provides an automated and efficient assessment of multiple EF components (http://www.cambridgecognition.com). Due to its normative procedures and nonverbal nature, CANTAB is highly suitable for use in different cultural settings (De Luca et al., 2003; Gau and Shang, 2010). CANTAB had been used extensively to assess cognitive impairments of patients with mental illness (Ozonoff et al., 2004; Gau and Shang, 2010; Collinson et al., 2014), and group differences in EF (De Luca et al., 2003).

In the present study, four cognitive tests were selected from the CANTAB to measure EF including Stockings of Cambridge (SOC), Spatial Working Memory (SWM), Spatial Span (SSP), and Intra-dimensional/Extra-dimensional Shifts (IED). Before each test, participants were told the rules through a brief oral instruction from the experimenter to ensure an accurate understanding. Participants were tested individually in a quiet room. All tests were performed on a computer screen.

The SOC was designed to be similar to Tower of London tasks, which assessed the ability of spatial planning and motor control. Two groups of patterns containing three colored balls were displayed on the computer screen in a specific configuration. The participants needed to move the balls on the bottom of screen to match with the goal set on the top using as few moves as possible. The number of moves increased from 2 to 4. In the motor control phases inserted in the test, the software waited for 500 ms and then moved a ball in the example configuration, and the subject needed to follow what it did. The duration after each problem was 3000 ms. The outcome scores of problems solved in minimum moves were chosen to measure planning (Table 2).

**Table 2:** **The scores of EF performances provided by CANTAB**.

**EF tasks**	**Components of EF measurement**	**Outcome measures scores**	**Mean**	**SD**
SOC	planning	Problems solved in minimum moves	−1.26	1.07
SWM	working memory	Between errors	−0.58	1.34
		Strategy	−0.53	0.92
SSP	short−term memory	Span length	0.54	1.11
		Total errors	−0.14	1.34
IED	inhibition	Pre−ED errors	0.05	0.59
	switch	EDS errors	−0.36	1.23

*SOC, Stockings of Cambridge; SWM, Spatial Working Memory; SSP, Spatial Span; IED, Intra-dimensional/Extra-dimensional Shifts. The right two columns indicate the mean value and standard deviation (SD) of the scores from 90 participants, respectively*.

The SWM assessed the ability to retain spatial information and to manipulate remembered items in working memory and heuristic strategy. The test began with a specific number of colored squares (boxes) shown on the screen. Participants were required to find one blue “token” in each of the square through the boxes with an increasing number (3 to 8). When a blue token was found, it would be used to fill up an empty column on the right hand side of the screen. The reveal time for the empty content of the box was 1000 ms. The outcome scores of “between errors” and strategy were chosen to measure working memory (Table 2).

The SSP measured the capacity of spatial short-term memory. In this task, nine white boxes were displayed on the screen at the beginning and then some of them would change color in a specific order. Participants were asked to repeat the order by clicking the boxes which had changed color. The difficulty level ranged from 2 to 9 boxes. The stimulus duration of color changing was 3000 ms, and the inter-stimulus time was 500 ms. After the last box in the sequence had reverted to white, there would be a delay of 1000 ms followed by a beep with a duration of 1000 ms (interim sound duration). The outcome scores of span length and total errors were chosen to measure short-term memory (Table 2).

The IED assessed the ability of attention set shifting which included inhibition and switch. The participants needed to determine the rules by clicking on one of the two colored graphics randomly in each trial and make the right choice according to the feedback of computer. The rules would change in a new stage (after six trials of consecutive right choices). If the participant still did not meet the criterion above after 50 trials at any stage, the test was terminated. These transformations included a reinforced stimulus dimension (intra-dimensional shift) corresponding to the ability of inhibition, and a previously irrelevant stimulus dimension (extra-dimensional shift) corresponding to the ability of switch. There was a pause of 250 ms (pre-stimulus pause) before the test stimuli were added to the boxes. The feedback of choice was displayed for 1500 ms in each trial, and following the feedback there was a blank screen with a duration of 1000 ms. The outcome scores of errors made prior to the extra-dimensional shift of the task (Pre-ED errors) were chosen to measure inhibition, and the outcome scores of errors made in the extra-dimensional shift stage (EDS errors) were chosen to measure switch (Table 2).

The correlation between each of component of EF score was listed in Table 3.

**Table 3:** **The correlation between each of component of EF score**.

**Components of EF**	**planning**		**working memory**		**short-term memory**		**inhibition**		**switch**	
	***R***	***p***	***R***	***p***	***R***	***p***	***R***	***p***	***R***	***p***
planning	—		**0.234**	**0.026**	0.110	0.303	−0.056	0.602	0.072	0.499
working memory	—		—		0.016	0.882	0.022	0.836	−0.034	0.748
short-term memory	—		—		—		0.080	0.454	0.032	0.762
inhibition	—		—		—		—		−0.025	0.815
switch	—		—		—		—		—	

*The bold values represent the correlation coefficient and the corresponding p-value with significant correlation*.

### Construction of the brain functional networks

The complex brain network analysis was applied to our resting-state data. The most critical elements of a complex network are nodes and edges. The nodes were defined as the positions of the 24 NIRS channels and the edges were defined as functional connectivity between the node pairs. By calculating the Pearson correlation coefficients of time courses between each pair of nodes to quantify the functional connectivity, a 24 × 24 correlation matrix was obtained for each participant. Then each correlation matrix was converted to binary matrixes by applying fixed thresholds (network density). In this study, the thresholds were set over a wide network density range (10–46%) at the intervals of 1% because the wide density range could maintain the small-world properties (Watts and Strogatz, 1998; Tian et al., 2011).

### Network analysis

#### Evaluation of the small-world property

To evaluate whether our resting-state data indeed had the small-world properties, we first calculated our small-world network parameters to see whether they met the following criteria:

(1) $$\gamma =C_{net}/C_{ran}>1,\lambda =L_{net}/L_{ran}\approx 1\;and\;\sigma =\gamma /\lambda >1$$

where *C*_*net*_ and *L*_*net*_ represent the clustering coefficient and the characteristic path length [see basic definitions (4) and (5)] of our real networks, respectively, and *C*_*ran*_ and *L*_*ran*_ are the corresponding indices drawn from the average of 100 matched random networks (Watts and Strogatz, 1998; Maslov and Sneppen, 2002; Tian et al., 2011).

The small-world property of network could be summarized as: σ = γ/λ > 1. In this study, we carried out the statistical tests to further verify σ > 1. Specifically, for each of network threshold range (10–46%, at the intervals of 1%), we tested whether σ significantly was greater than 1 across all the participants using a one-sample *t*-test.

#### Network topological properties

We obtained seven network topological properties metrics including three regional nodal parameters (nodal degree *D*_*nod*_, nodal efficiency *E*_*nod*_, nodal betweenness centrality *N*_*bc*_), and four global parameters (clustering coefficient *C*_*p*_, characteristic path length *L*_*p*_, global efficiency *E*_*glob*_, local efficiency *E*_*loc*_) by using the graph theoretical network analysis toolbox: GRETNA (http://www.nitrc.org/projects/gretna/). Although there are many parameters to characterize network functional connectivity, we chose these particular parameters because they have been commonly used in the existing neuroimaging studies to characterize the global and regional nodal network properties (Li et al., 2009; Tian et al., 2011; Niu et al., 2012). Second and more specifically, some previous studies (Tian et al., 2011; Niu et al., 2012; Wang et al., 2012) have used these network parameters to characterize resting-state network functional connectivities. To ensure comparability between these existing network functional connectivity studies and the present study, we chose to use these specific parameters.

For a graph *G, N* represented the total number of nodes in the network. The basic definitions are listed as follows.

Nodal degree [*D*_*nod*_(*i*)] is the number of edges linked to the node *i*. *D*_*nod*_(*i*) reflectes the importance of node *i* in the network structure (Tian et al., 2011; Wang et al., 2012).

Nodal efficiency [*E*_*nod*_(*i*)] (Achard and Bullmore, 2007):

(2) $$E_{nod}(i)=\frac{1}{\left(N-1\right)}\sum_{j\;\neq \;i\in G}\frac{1}{L_{ij}}$$

where *L*_*ij*_ represents the shortest path length from node *i* to *j*. The *E*_*nod*_(*i*) measures the information transfer efficiency between the node *i* and other nodes (Wang et al., 2012).

Nodal betweenness centrality [*N*_*bc*_(*i*)] (Freeman, 1977):

(3) $$N_{bc}(i)\text{=}\sum_{j\;\neq \;i\;\neq \;k\in G}\frac{\delta_{jk}(i)}{\delta_{jk}}$$

where δ_*jk*_ represents the number of shortest paths from node *j* to node *k*, and δ_*jk*_(*i*) represents the number of shortest paths from node *j* to node *k* passing through node *i* within graph *G*. The *N*_*bc*_(*i*) reflects the importance of node *i* over information flow in the entire network (Tian et al., 2011; Xu et al., 2014).

Nodal degree measures the local interconnection capability of a brain region (Wang et al., 2012), nodal efficiency the transfer capability (Wang et al., 2012), and nodal betweenness centrality the frequency of participation in information transition of specific areas over the whole network (Freeman, 1977; Achard and Bullmore, 2007; Wang et al., 2012; Gao et al., 2013). These three regional parameters reflect the importance of the specialization and integration of information processing of specific brain areas in the whole functional network (Freeman, 1977; Achard and Bullmore, 2007; Li et al., 2009; Tian et al., 2011; Xu et al., 2014).

Clustering coefficient *C*_*P*_ (Watts and Strogatz, 1998):

(4) $$C_{P}=\frac{1}{N}\sum_{i\in G}\frac{2E_{i}}{D_{nod}(i)(D_{nod}(i)-1)}$$

where *E*_*i*_ is the number of edges in the subgraph *G*_*i*_ which consists of the neighbors of node *i*.

Characteristic path length *L*_*P*_ (Newman, 2003):

(5) $$L_{P}=\frac{1}{\frac{1}{N(N-1)}\left(\sum_{j\;\neq \;i\in G}\frac{1}{L_{ij}}\right)}$$

Global efficiency *E*_*glob*_ (Latora and Marchiori, 2001):

(6) $$E_{glob}=\frac{1}{N\left(N-1\right)}\sum_{j\;\neq \;i\in G}\frac{1}{L_{ij}}$$

Local efficiency *E*_*loc*_ (Latora and Marchiori, 2001):

(7) $$E_{loc}=\frac{1}{N}\sum_{i\in G}E_{glob}(i)$$

where *E*_*glob*_(*i*) is the global efficiency of *G*_*i*_.

*C*_*P*_ and *E*_*loc*_ measure the clustering degree of the entire network, whereas *L*_*P*_ and *E*_*glob*_ measure the global information transfer efficiency of a network with the former being the inverse of the latter (Tian et al., 2011).

We calculated the integral quantity to obtain the summations of each network density for every participant (Tian et al., 2011). For the global network parameters,

(8) $$P_{glob}=\sum_{k\;=\;10}^{46}P(k\Delta s)\Delta s$$

where Δ*s* is the density interval of 1%; *P*(*kΔs*) represents the global network parameter at the network density of *kΔs*.

For the regional nodal parameters:

(9) $$P_{nod}(i)=\sum_{k\;=\;10}^{46}P(i,k\Delta s)\Delta s$$

where *P*(*i, kΔs*) represents a regional nodal parameter of the node *i* at the network density of *kΔs*.

### Correlation analyses

We used scores produced by each of the four tasks in CANTAB as the raw scores. All raw scores were then converted to z-scores before further analyses:

(10) $$Z=\frac{\left(X-M\right)}{S}$$

where *X* is the raw scores, *M* is the mean value of the raw scores from the entire sample, and *S* is the standard deviation of the raw scores from the entire sample.

Because the working memory task produced two raw scores (i.e., between errors and strategy; Table 2), we first normalized each of these two raw scores, and then added up these two normalized scores as an index of their performance of working memory. We did the same for the short-term memory task, namely that we added up the normalized scores of span length and total errors as an index of their performance of the short-term memory task. With respect to the two raw scores of IED, they actually indicated two performances (i.e., inhibition and switch), respectively. We therefore only normalized each of these two raw scores. Thus, in total, we obtained five scores: planning, working memory, short-term memory, inhibition, and switch (Table 2). Additionally, we added up all of these five normalized scores to obtain the Total EF score.

Then, to examine the linkage between resting-state functional neutral network topological properties and EF performances, we performed correlation analyses of neural network topological property and behavioral EF scores with the permutations tests. We first computed correlation coefficient of each EF score with each network topological index. Then, we performed a permutation test to assess the statistical significance of the coefficient.

Below we used Total EF score and nodal degree to illustrate how such test was implemented.

1. We obtained a Pearson correlation coefficient between Total EF score and nodal degree with the data from all 90 participants to obtain a non-permuted correlation coefficients.
2. Total EF scores were scrambled (randomly permuted) and were correlated with nodal degree (unscrambled) to obtain a permuted Pearson correlation coefficient. We did so 1000 times.
3. Based on (2), we ordered the permuted correlation coefficients according to their values from the lowest to the highest. We obtained the 25th values from both the top and bottom of the distribution to establish 95% bilateral confidence intervals. The *p* values are defined as (Tian et al., 2016):
  (11) $$p=\frac{\text{1 }+\;N_{greater}}{\text{1 }+\;N}$$
  Where *N* is the number of permutations, and here *N* = 1000; *N*_*greater*_ is the number of permuted correlation coefficients whose absolute value is greater than that of absolute value of non-permuted correlation coefficients. If *p* < 0.05, the non-permuted correlation coefficient is considered significant.

## Result

### Small-world properties of network

**Figure 2:**
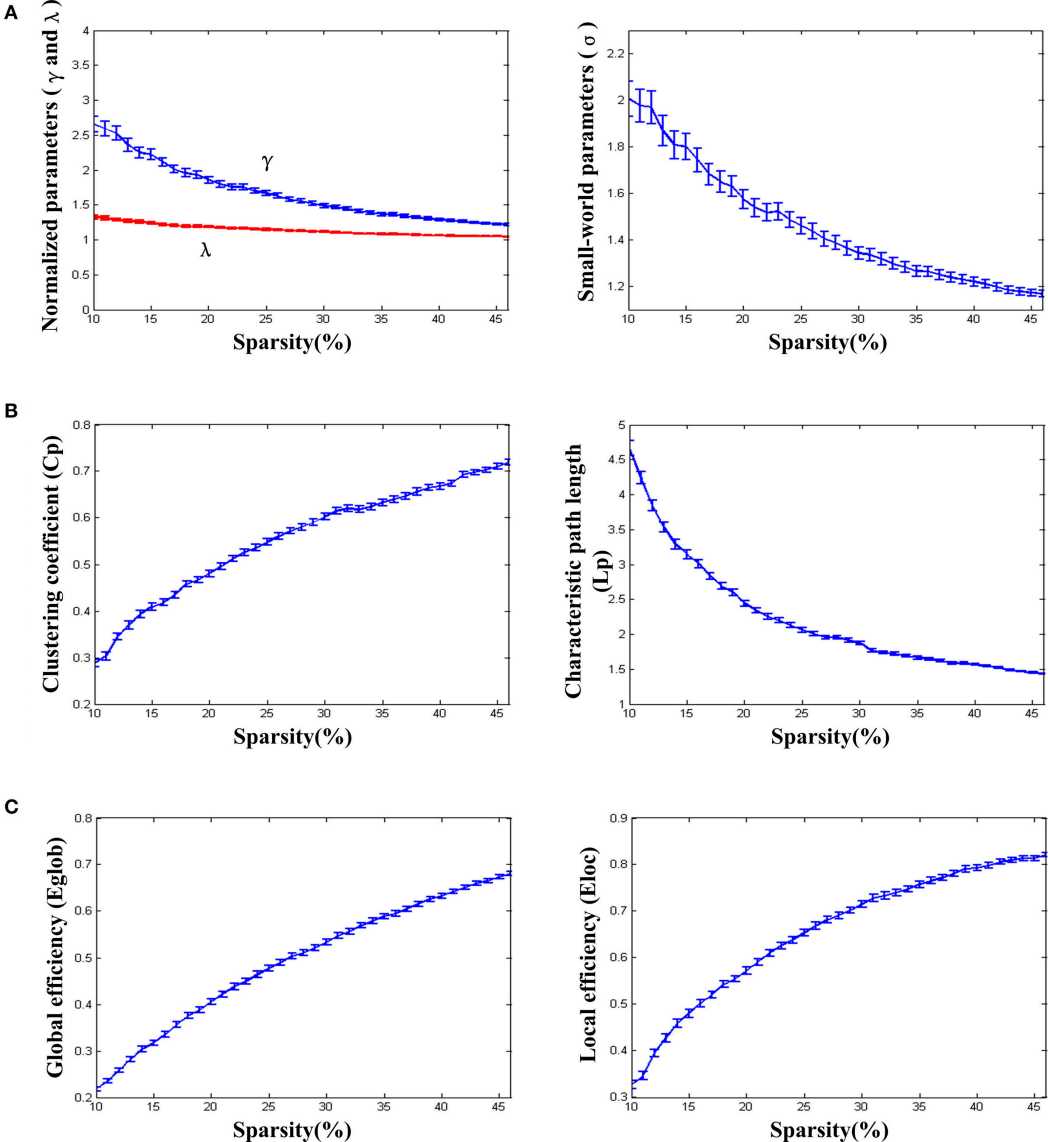
**The small-world properties and efficiency of the resting-state functional network**. **(A)** Normalized parameters (γ and λ) and small-world parameters (σ). **(B)** Clustering coefficient (*C*_*p*_) and characteristic path length (*L*_*p*_). **(C)** Global efficiency (*E*_*glob*_) and local efficiency (*E*_*loc*_). The standard errors were shown on the figures. The parametric statistical values were averaged over the networks of all individuals (removing abnormal data based on the triple standard difference method).

**Figure 3:**
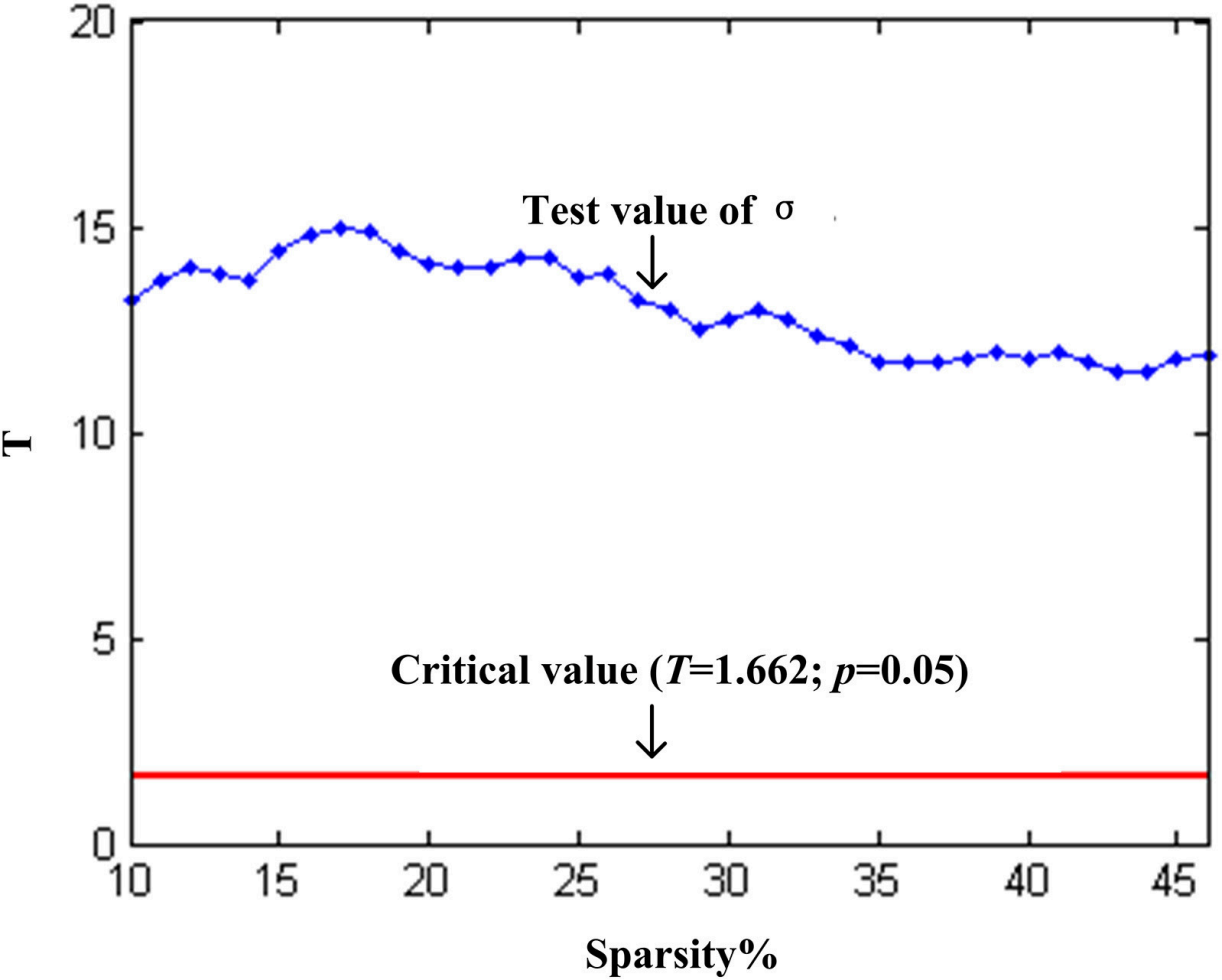
**One-sample *t*-test statistics of σ (compared to 1) at each network threshold**. The vertical axis is the t-statistic values of the one-sample *t*-test. The blue line represents the t-statistic values of σ at each network threshold, and the red line represents the t-statistic critical value (*T* = 1.662) at the significant level of *p* = 0.05 (one-tailed).

We found that over the network density range of 10–46%, γ was greater than 1, λ was approximately equal to 1, σ was greater than 1 and decreased gradually with the increase of density (Figure 2A). One-sample *t*-test showed that the t-statistic values of σ was far greater than the t-statistic critical value at the significant level of *p* < 0.05 (one-tailed) (Figure 3). The result verified σ>1 of the networks in our study. Thus, the functional network in the PFC showed prominent small-world properties similar to those with the whole brain functional network reported in the previous studies (Tian et al., 2011; Niu et al., 2012). Additional results (*C*_*P*_, *L*_*P*_, *E*_*glob*_, *E*_*loc*_) (Figures 2B,C) further supported the small-world properties, which integrated the high information processing efficiency and local connectivity effectively (Watts and Strogatz, 1998; Li et al., 2009; Tian et al., 2011; Niu et al., 2012).

### Relations between network properties and behavioral EF scores

The behavioral results of EF performances included scores of Total EF, planning, working memory, short-term memory, inhibition, and switch provided by CANTAB (Table 2).

None of the behavioral EF scores were correlated with any of the global network topological parameters. However, the behavioral EF scores were significantly correlated with several nodal network topological parameters.

**Figure 4:**
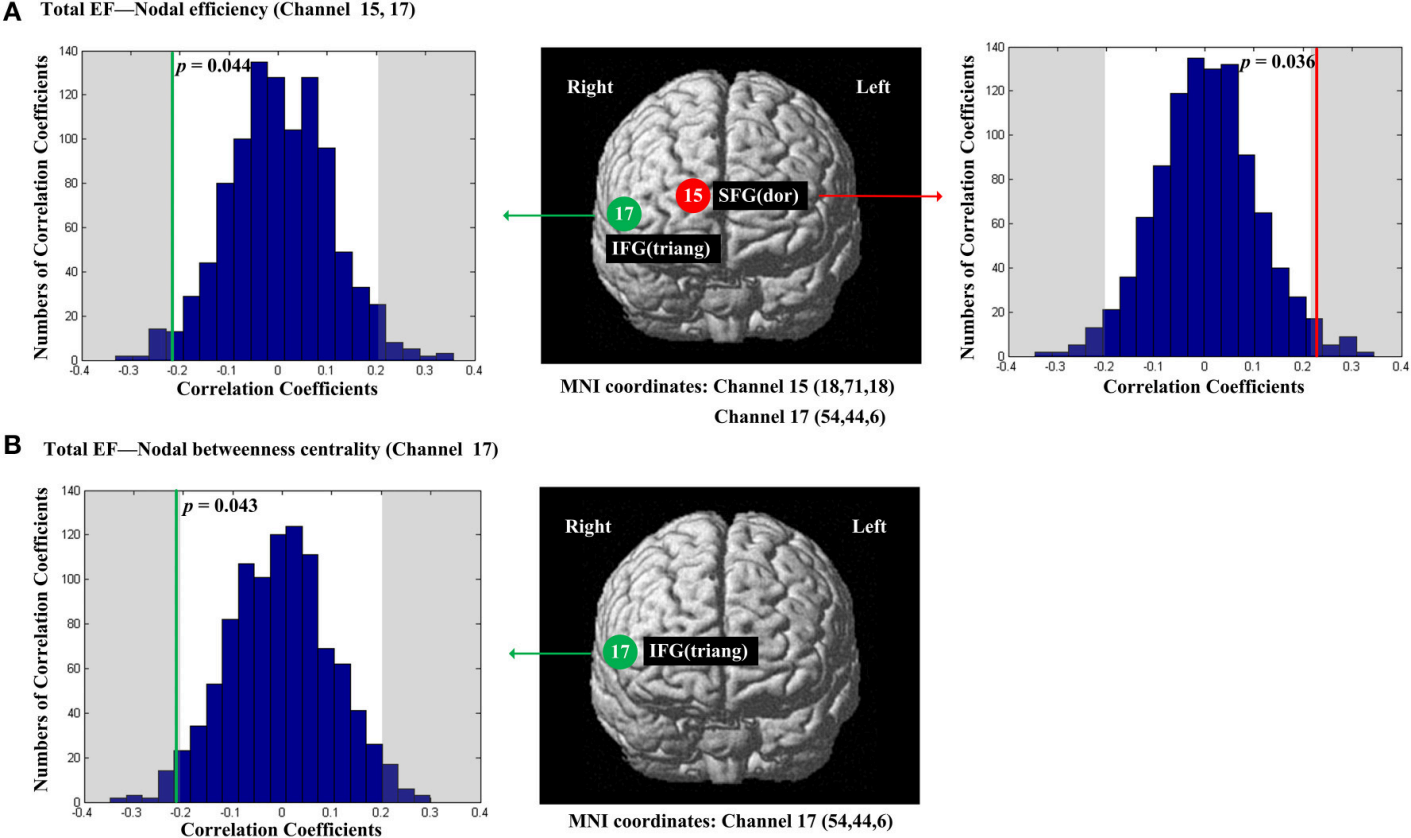
**The estimated positions of the cortical regions showing significant correlation between the scores of Total EF and the regional nodal topological properties of the network**. **(A)** Total EF and nodal efficiency. **(B)** Total EF and nodal betweenness centrality. These positions are labeled using channel number in red circles (positive correlation) or green circles (negative correlation) with respective MNI coordinates shown below. The left and right subgraphs show the permutations tests results of correlation coefficients. The shaded parts are the reject regions of permutations tests at significant level 5% (two-tailed) and the *p*-*values* are shown in the subgraphs. The red line and green lines indicate the positive and negative non-permuted correlation coefficients, respectively. Abbreviation: SFG (dor), dorsal superior frontal gyrus; IFG (triang), triangular inferior frontal gyrus.

The scores of Total EF were significantly and positively correlated with nodal efficiency (*E*_*nod*_) in the right SFG (*p* = 0.036, channel 15, BA 10; Figure 4A); The scores of Total EF were also significantly and negatively correlated with nodal efficiency (*E*_*nod*_) the right IFG (*p* = 0.044, channel 17, BA 45; Figure 4A), and nodal betweenness centrality (*N*_*bc*_) in the right IFG (*p* = 0.043, channel 17, BA 45; Figure 4B).

**Figure 5:**
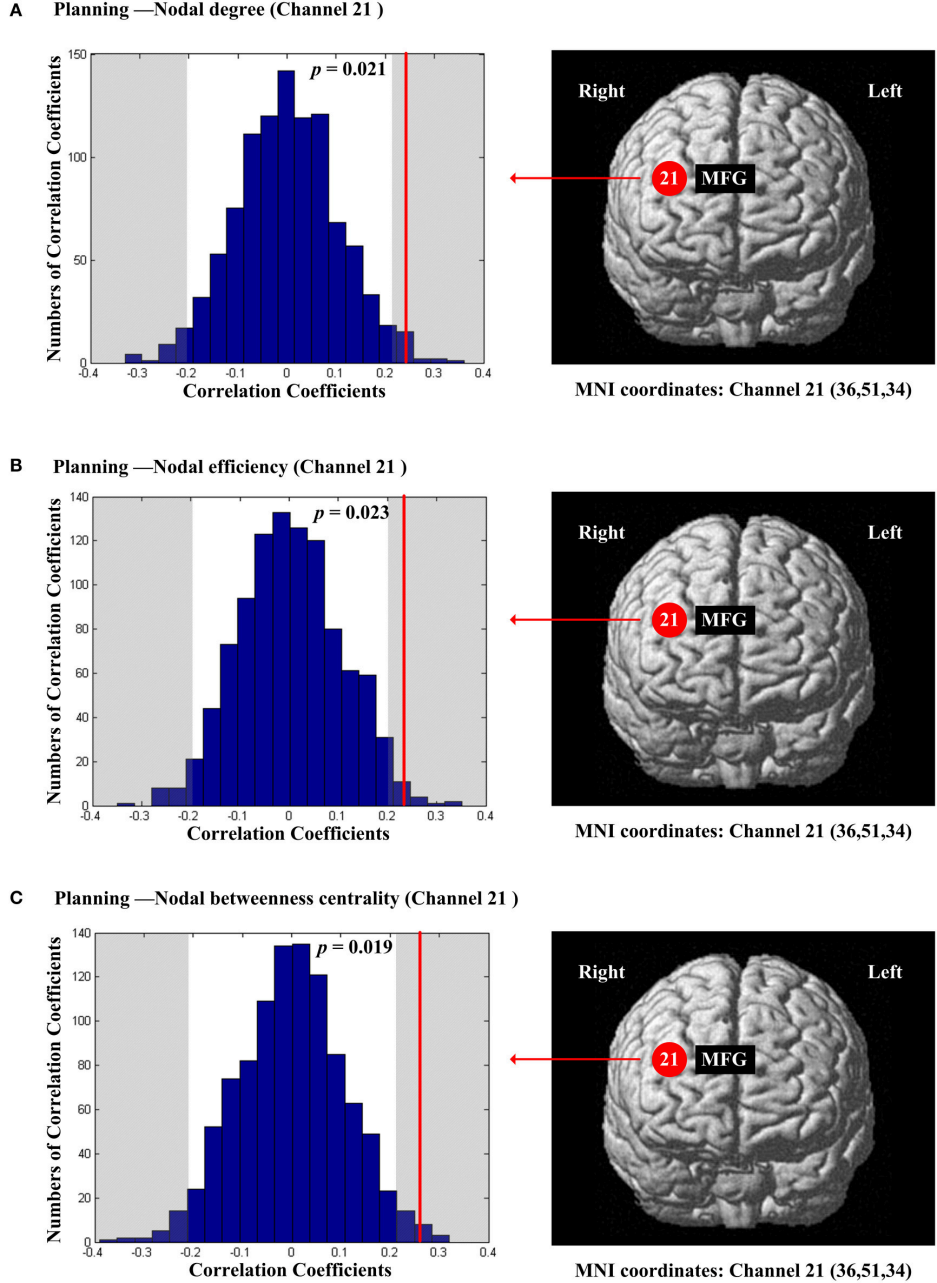
**The estimated positions of the cortical regions showing significant correlation between the scores of planning and the regional nodal topological properties of the network**. **(A)** Planning and nodal degree. **(B)** Planning and nodal efficiency. **(C)** Planning and nodal betweenness centrality. These positions are labeled using channel number in red circles (positive correlation) with respective MNI coordinates shown below. The left subgraphs show the permutations tests results of correlation coefficients. The shaded parts are the reject regions of permutations tests at significant level 5% (two-tailed) and the *p*-*values* are shown in the subgraphs. The red line indicates the positive non-permuted correlation coefficients. Abbreviation: MFG, middle frontal gyrus.

**Figure 6:**
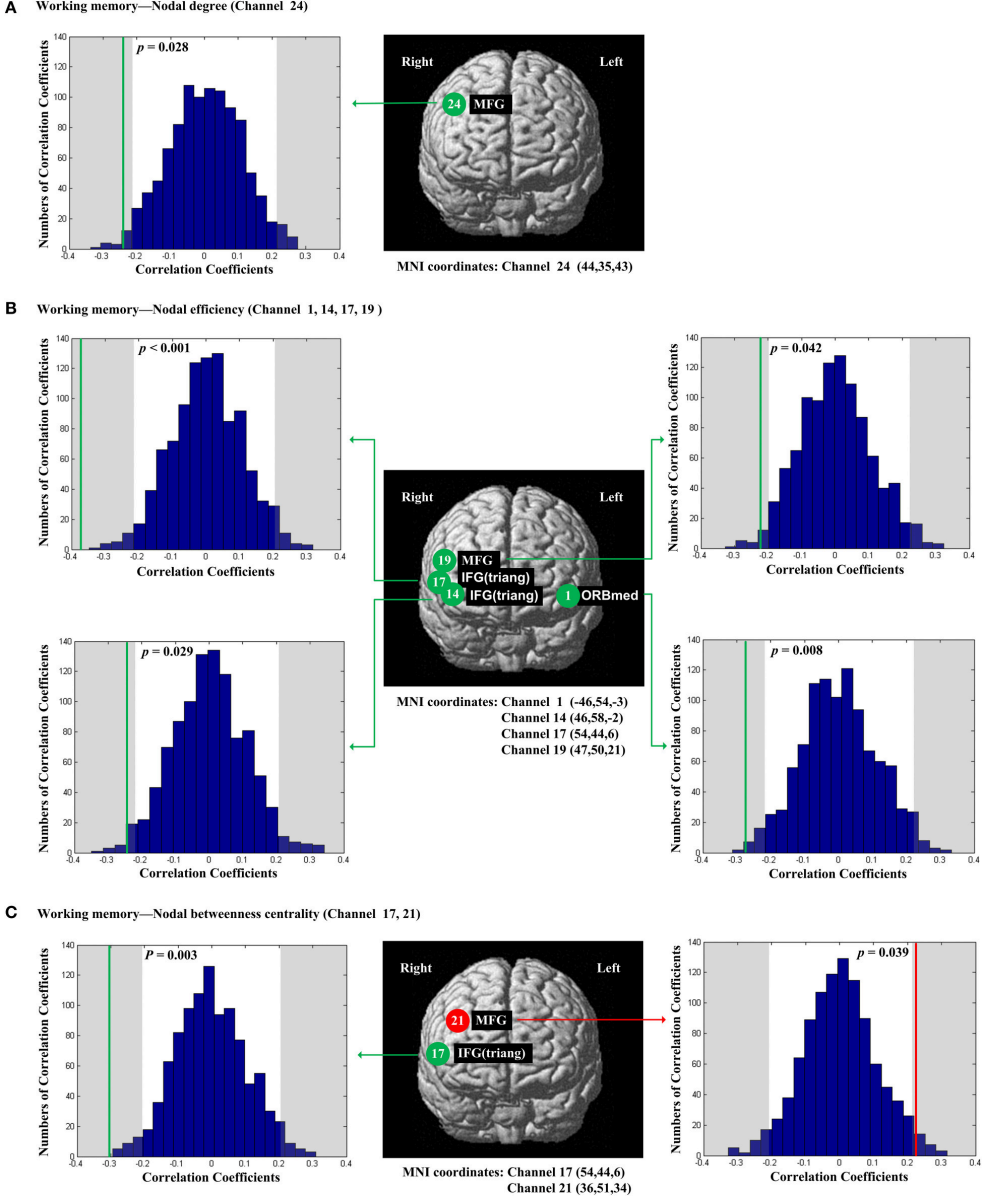
**The estimated positions of the cortical regions showing significant correlation between the scores of working memory and the regional nodal topological properties of the network**. **(A)** Working memory and nodal degree. **(B)** Working memory and nodal efficiency. **(C)** Working memory and nodal betweenness centrality. These positions are labeled using channel number in red circles (positive correlation) or green circles (negative correlation) with respective MNI coordinates shown below. The left and right subgraphs show the permutations tests results of correlation coefficients. The shaded parts are the reject regions of permutations tests at significant level 5% (two-tailed) and the *p*-*values* are shown in the subgraphs. The red line and green lines indicate the positive and negative non-permuted correlation coefficients, respectively. Abbreviation: MFG, middle frontal gyrus; ORBmed, Orbitofrontal cortex (medial); IFG (triang), triangular inferior frontal gyrus.

**Figure 7:**
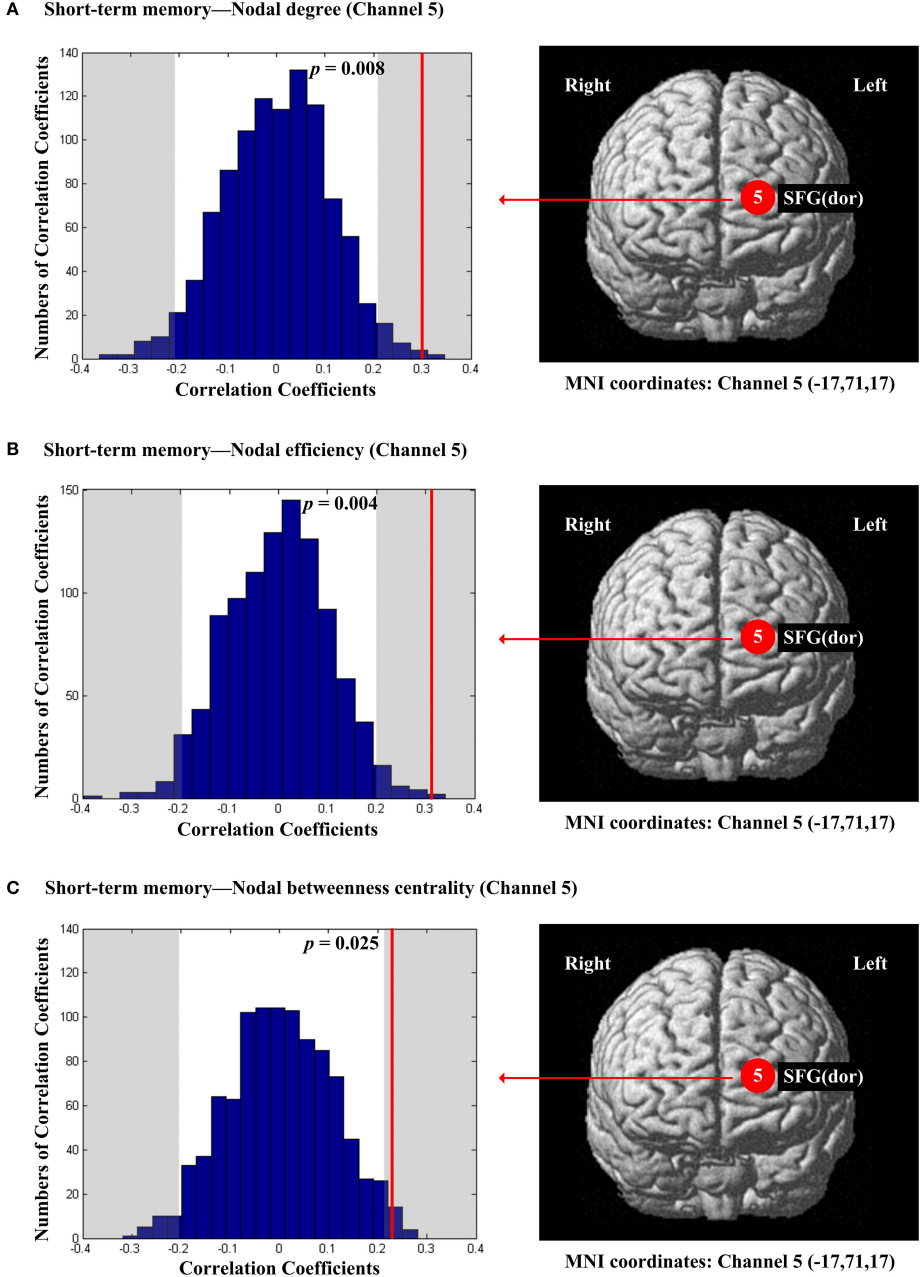
**The estimated positions of the cortical regions showing significant correlation between the scores of short-term memory and the regional nodal topological properties of the network**. **(A)** Short-term memory and nodal degree. **(B)** Short-term memory and nodal efficiency. **(C)** Short-term memory and nodal betweenness centrality. These positions are labeled using channel number in red circles (positive correlation) with respective MNI coordinates shown below. The left subgraphs show the permutations tests results of correlation coefficients. The shaded parts are the reject regions of permutations tests at significant level 5% (two-tailed) and the *p*-*values* are shown in the subgraphs. The red line indicates the positive non-permuted correlation coefficients. Abbreviation: SFG (dor), dorsal superior frontal gyrus.

The scores of planning were significantly and positively correlated with nodal degree (*D*_*nod*_, *p* = 0.021; Figure 5A), nodal efficiency (*E*_*nod*_, *p* = 0.023; Figure 5B) and nodal betweenness centrality (*N*_*bc*_, *p* = 0.019; Figure 5C) in the right MFG (channel 21, BA 46). The scores of working memory were significantly and negatively correlated with nodal degree (*D*_*nod*_) in the right MFG (*p* = 0.028, channel 24, BA 9; Figure 6A), nodal efficiency (*E*_*nod*_) in the left orbital MFG (*p* = 0.008, channel 1, BA 46), the right IFG (*p* = 0.029, channel 14, BA 46), the right IFG (*p* < 0.001, channel 17, BA 45) and the right MFG (*p* = 0.042, channel 19, BA 46; Figure 6B), and nodal betweenness centrality (*N*_*bc*_) in the right IFG (*p* = 0.003, channel 17, BA 45; Figure 6C). The scores of working memory were also significantly and positively correlated with the right MFG (*p* = 0.039, channel 21, BA 46; Figure 6C). The scores of short-term memory were significantly and positively correlated with nodal degree (*D*_*nod*_, *p* = 0.008; (Figure 7A), nodal efficiency (*E*_*nod*_, *p* = 0.004; Figure 7B) and nodal betweenness centrality (*N*_*bc*_, *p* = 0.025; Figure 7C) in the left SFG (channel 5, BA 10).

**Figure 8:**
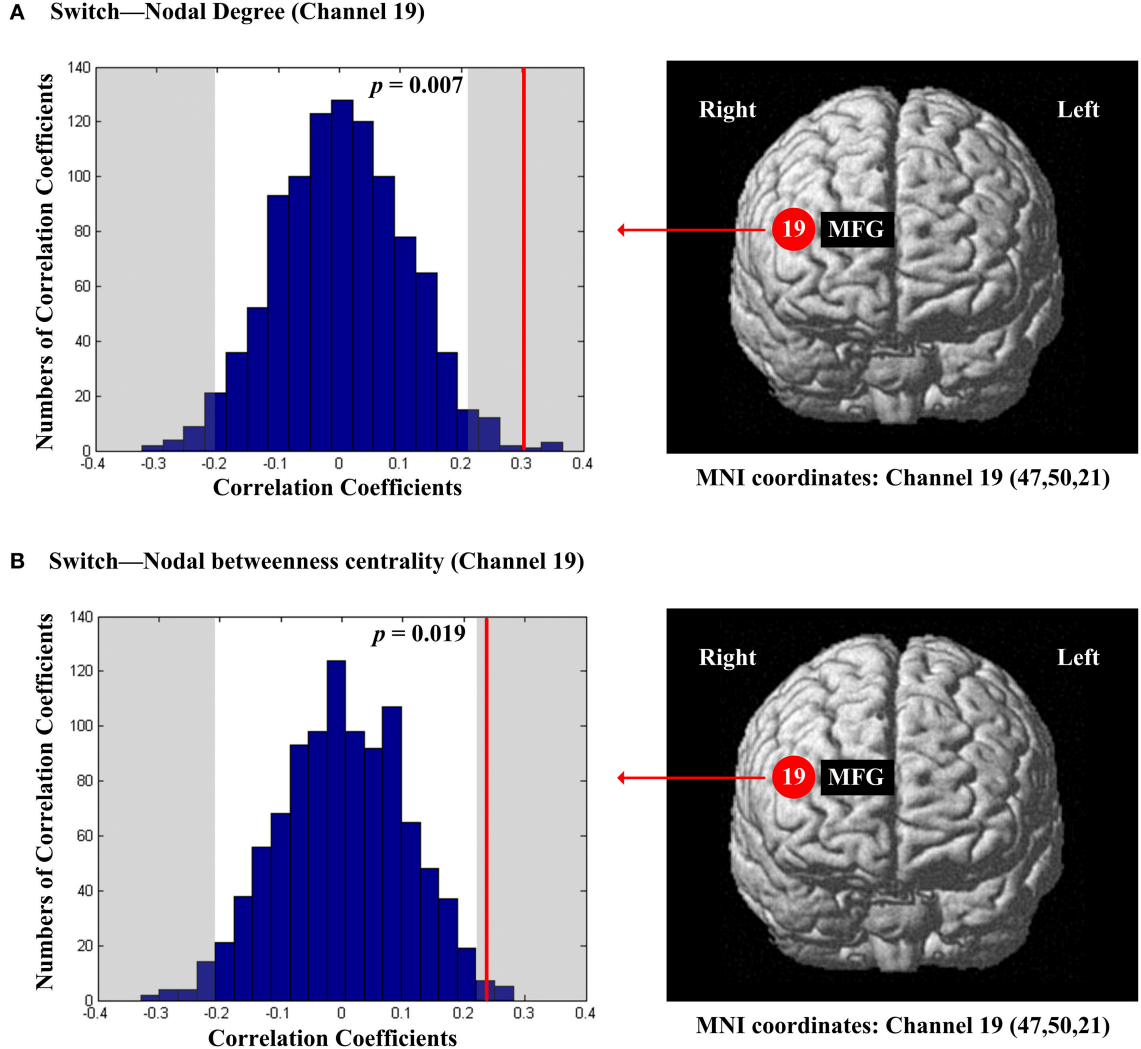
**The estimated positions of the cortical regions showing significant correlation between the scores of switch and the regional nodal topological properties of the network**. **(A)** Switch and nodal degree. **(B)** Switch and nodal betweenness centrality. These positions are labeled using channel number in red circles (positive correlation) with respective MNI coordinates shown below. The left subgraphs show the permutations tests results of correlation coefficients. The shaded parts are the reject regions of permutations tests at significant level 5% (two-tailed) and the *p*-*values* are shown in the subgraphs. The red line indicates the positive non-permuted correlation coefficients. Abbreviation: MFG, middle frontal gyrus.

The scores of inhibition were not significantly correlated with any of the regional nodal network topological parameters. The scores of switch were significantly and positively correlated with nodal degree (*D*_*nod*_, *p* = 0.007; Figure 8A) and nodal betweenness centrality (*N*_*bc*_, *p* = 0.019; Figure 8B) in the right MFG (channel 19, BA 46).

## Discussion

In this study, we investigated the relations between behavioral performance in various EF tasks and the fNIRS resting-state functional network global and local topological properties in the PFC. We obtained several major findings.

### Small-world network properties

We found prominent small-world properties in our participants' resting-state brain functional networks. This finding is consistent with the results of several fMRI resting-state studies (Achard et al., 2006; Tian et al., 2011). More specifically, our findings are in line with an existing fNIRS resting-state study (Niu et al., 2012) reporting that the whole brain resting-state network has prominent small-world network properties. The present finding taken together with those from Niu et al. (2012) suggested that small-world properties are the essential characteristics of brain resting-state network. Further, our finding suggested that fNIRS is an effective technology to describe the topological properties of the brain functional network.

### Relations between behavioral EF scores and neural network parameters

We found that none of the four global parameters (i.e., clustering coefficient, characteristic path length, global efficiency and local efficiency) was related to any of the behavioral EF scores. However, EF scores were significantly correlated with the regional nodal network parameters (i.e., nodal degree, nodal efficiency and nodal betweenness centrality). These findings support the Local Property Only Hypothesis.

Our findings are thus in contrast to existing studies that examined the relations between intelligence and the network properties of the resting-state functional connectivity in healthy adults (van den Heuvel et al., 2009; Langer et al., 2012). They found that intelligence was significantly correlated with the global network indexes. Our findings taken together with these existing studies suggested that the global network properties may underlie such a general ability as intelligence, whereas the network regional properties may subserve such a special cognitive ability as EF. In other words, the specific aspects of the network properties of the resting-state brain network play different roles for different cognitive abilities. As EF involves a specialized set of cortical regions and the connections among them, the regional quality of the resting-state network matters more than its global quality.

This interpretation is consistent with our findings that Total EF scores and different components of EF are associated with the regional network properties in different cortical areas. For the Total EF scores, the better nodal efficiency the higher the Total EF scores in the right dorsal SFG, whereas the opposite pattern was observed in the right triangular IFG (the better nodal efficiency and nodal betweenness centrality the lower Total EF scores; Figure 4). The opposite patterns of the Total EF scores might reflect the different roles of SFG and IFG in EF (Petrides, 2005). A recent fMRI study (Reineberg et al., 2015) examined the relations between Total EF scores and resting-state functional connectivity. They found that the Total EF was associated with functional connectivity intensity of the right frontoparietal resting-state functional network, which was consistent with our findings. We found that better information transfer of SFG was associated with higher Total EF scores, and better information transfer of IFG was linked to lower Total EF scores. Although the specific functions of SFG and IFG for EF are yet to be ascertained, our findings support the idea that the right PFC in the resting-state is crucial to EF overall.

For planning, we found that the ability of planning was positively correlated with all the three resting-state regional network parameters in the right MFG (BA46) (Figure 5). A study by Woo et al. (2010) using positron emission tomography (PET) showed that poor planning performances in patients with Alzheimer's disease were associated with lower resting metabolism in the right MFG and adjacent IFG (BA45 and BA46). Woo et al. (2010) suggested that the right PFC was necessary to foster planning ability that was consistent with our findings. Moreover, a previous task-based fMRI study (Newman et al., 2003) showed that although both the right and left PFC were activated by a planning task, the activation in the right dorsolateral prefrontal cortex (DLPFC) was attenuated by task difficulty. Newman et al. (2003) confirmed that the right PFC areas were involved in information generation and integration of planning and strategy. In addition, Petrides, (2005) suggested the middle DLPFC (BA 46, and 9/46) could control cognition and planned behavior consciously. Our findings further sugested that the right MFG may be a central area for the planning ability with both high local association capability and high information transfer level. In other words, the resting-state network quality of the right MFG may play a key role in performing organizational planning functions during cognitive processes (Woo et al., 2010).

For working memory, the score of spatial working memory was significantly correlated with nodal degree in the right MFG (BA 9) (Figure 6A). The score of working memory was also significantly correlated with the nodal efficiency and nodal betweenness centrality in parts of the right triangular IFG and the adjacent MFG (BA 45 and 46) (Figures 6B,C). In addition, working memory was significantly correlated with nodal efficiency of the left orbitofrontal cortex (BA46) (Figure 6B). Our findings are generally consistent with the findings of a recent study by Zou et al. (2013). They found that the amplitude of low-frequency fluctuation (ALFF) in the MFG was related to the task-evoked activation associated with working memory. Given the fact that the scores of planning and those of working memory were significantly correlated (*r* = 0.23; *p* = 0.026; Table 3), our findings suggested that the MFG may be involved in information processing underlying both planning and working memory. Additionally, based on the negative correlations in our results in the right triangular IFG and left orbitofrontal cortex, the high internally spontaneous activity levels, especially information transfer levels of these areas of PFC, may be detrimental to one's working memory.

The significant positive correlations between the score of short-term memory and all the three resting-state regional network characteristics were localized in the left SFG (BA10) (Figure 7). An fMRI study by Wu et al. (2014) showed that patients with amnestic mild cognitive impairment indicated both significantly declined short-term memory and reduced resting-state connectivity strength in the bilateral DLPFC. In particular, among the findings of Wu et al. (2014), the functional connectivity of the left SFG (BA 10) was significantly reduced, consistent with our results. Our findings support the relationship between the spontaneous activity in the left frontal pole and the ability of short-term memory. In addition, combining with the results of working memory, it suggested that the different component of memory function involved different areas of PFC in resting-state.

The EF scores for inhibition showed no significant correlation with any of the regional nodal network topological parameters. In contrast, the EF scores for switch were significantly positively correlated with the nodal degree and nodal betweenness centrality in the right MFG (BA 46) (Figure 8). This finding suggested that the increased capabilities of both local association and frequency of information transition of the right MFG (BA 46) are linked to the increased switch level. Our results are not entirely in line with the existing studies that examined the activations of cortical regions involved in performing actual inhibition and switch tasks. For example, the existing studies examining neural correlates of actual inhibition revealed the activation of IFG specifically (Aron et al., 2004; Collette et al., 2006; Vidal et al., 2012). For switch, several studies found that it was associated with the DLPFC broadly (Collette et al., 2006; Jazbec et al., 2007). Moreover, the different cortical areas of PFC are activated during different stages of the switch task (Oh et al., 2014). As the right MFG (BA 46) was also associated with planning and working memory (see above), the middle DLPFC might be a crucial area for the information integration and controlling of EF (Petrides, 2005). The discrepancy between our resting-state findings and the existing task-evoked findings may be due to multiple reasons, such as the differences in research foci (our study's focus on network properties vs. the existing research's focus on neural activations) and the task demands (resting-state vs. performing a specific task). Future specifically designed studies could address this issue by linking the resting-state network properties to both EF behavioral performance and EF task related activations.

### Limitation and conclusion

Despite our novel findings, the limitations should be taken into consideration. First, in this study, we only assessed the network properties in the PFC regions. Although our findings are in line with the existing studies that suggest the central role of the prefrontal cortex (PFC) in EF, recent studies suggested that some other regions, such as parietal cortex are also involved in EF. Thus, future studies may wish to extend the range of a similar fNIRS study to cover the whole cortical surface. Second, intelligence was not included in this study. It is generally accepted that intelligence is related to EF (Friedman et al., 2006; Duan et al., 2010) but the relation between them in the brain is less clear. Hence, it is necessary to introduce intelligence as a control variable to take into consideration the effect of intelligence on EF performance and its relation to the neural network topological properties. Third, changes in functional connectivity between two regions may also be affected by changes in signal or noise in either region or by another connection (Friston, 2011). Thus, future studies must use stricter experimental controls, a larger sample size, and more advanced statistical methods to rule out the influence of these potentially confounding factors. For example, future work employing a executive function task could utilize psychophysiological interaction analysis, which reveals consistent and specific patterns of effective connectivity (e.g., Smith et al., 2016). Forth, on one hand, the limited spatial resolution of fNIRS compared to fMRI may result in the less accurate space localization of the cortex regions. On the other hand, the superior temporal resolution of fNIRS is more conducive to describe the time course of the resting state fluctuations of the brain's neural activities. Thus, to study the resting-state neural correlation of EF, future studies should consider combining fNIRS with fMRI.

This study investigated the relationship between the behavioral EF scores and the resting-state functional network topological properties in the PFC utilizing functional near infrared spectroscopy (fNIRS). We found that only regional nodal but not global network properties were associated with EF components scores. We also observed that the different EF components were related to different regional properties in various PFC areas. Our findings suggested that the resting-state PFC activity plays an important role in individuals' behavioral performance in the executive function tasks. Further, the resting-state functional network can reveal the intrinsic neural mechanisms underlying behavioral EF abilities.

## Author contributions

Experimental design: JL, GF, KL, and XD. Experimental data recording: JZ, GZ, XJ, and GC. Experimental data analyze: JZ and JL. Manuscript writing: JZ and JL. Manuscript revision: JL and KL.

### Conflict of interest statement

The authors declare that the research was conducted in the absence of any commercial or financial relationships that could be construed as a potential conflict of interest.
